# Defective flow space limits the scaling up of turbulence bioreactors for platelet generation

**DOI:** 10.1038/s44172-024-00219-y

**Published:** 2024-06-17

**Authors:** Haruki Okamoto, Kosuke Fujio, Sou Nakamura, Yasuo Harada, Hideki Hayashi, Natsumi Higashi, Atsushi Ninomiya, Ryota Tanaka, Naoshi Sugimoto, Naoya Takayama, Atsushi Kaneda, Akira Sawaguchi, Yoshikazu Kato, Koji Eto

**Affiliations:** 1 Department of Clinical Application, Center for iPS Cell Research and Application (CiRA), Kyoto University, Kyoto, Japan; 2 Otsuka Pharmaceutical Company Limited, Department of Drug Modality Development, Osaka Research Center for Drug Discovery, Minoh, Osaka, Japan; 3 Department of Regenerative Medicine, Chiba University Graduate School of Medicine, Chiba, Japan; 4 Department of Molecular Oncology, Graduate School of Medicine, Chiba University, Chiba, Japan; 5 Department of Anatomy, Faculty of Medicine, University of Miyazaki, Miyazaki, Japan; 6 Mixing Technology Laboratory, SATAKE MultiMix Corporation, Toda, Saitama, Japan

**Keywords:** Regenerative medicine, Biologics

## Abstract

To complement donor-dependent platelets supplies, we previously developed an ex vivo manufacturing system using induced pluripotent stem cell (iPSC)-derived expandable immortalized megakaryocyte progenitor cell lines (imMKCLs), and a turbulent flow bioreactor to generate iPSC-derived platelets products (iPSC-PLTs). However, the tank size of the bioreactor was limited to 10 L. Here we examined the feasibility of scaling up to 50 L with reciprocal motion by two impellers. Under optimized turbulence parameters corresponding to 10 L bioreactor, 50 L bioreactor elicited iPSC-PLTs with intact in vivo hemostatic function but with less production efficiency. This insufficiency was caused by increased defective turbulent flow space. A computer simulation proposed that designing 50 L turbulent flow bioreactor with three impellers or a new bioreactor with a modified rotating impeller and unique structure reduces this space. These findings indicate that large-scale iPSC-PLTs manufacturing from cultured imMKCLs requires optimization of the tank structure in addition to optimal turbulent energy and shear stress.

## Abstract (web summary)

Haruki Okamoto and colleagues demonstrate that tank structure is crucial for large-scale platelet production from cultured immortalized megakaryocyte progenitor cell lines, due to the increase in defective turbulent flow space. These insights could guide the development of new bioreactors with improved production efficiency.

## Introduction

Platelets (PLTs) transfusions are given to patients to prevent bleeding in thrombocytopenia caused by conditions such as hematological diseases, cardiac surgery, and massive trauma. In the past 10 years, there has been increased demand for PLTs transfusions worldwide due to aging populations accompanied with advances in medical procedures/treatments^1–3^. However, the supply of donors has not kept pace^4,5^. In addition, patients allosensitized through PLTs transfusion or pregnancy can develop PLTs transfusion refractoriness, leaving them in need of human leukocyte antigen class I- and/or human PLTs antigen compatible donors, who can be difficult to find in cases of emergencies and rare types^5–7^. Therefore, several groups, including ours have proposed human induced pluripotent stem cells (iPSCs) as an alternative source, but the practical ex vivo manufacturing of 200–300 billion PLTs, which is the number used for one transfusion, has not been achieved until recently^8–12^.

We have shown that immortalized megakaryocyte cell lines (imMKCLs) derived from human iPSCs can be robustly expanded owing to the overexpression of c-MYC, BMI1, and BCL-XL, and that doxycycline (Dox) can be used to regulate the proliferation (Dox-ON) and maturation (Dox-OFF) of these cells^13^. Meanwhile, based on in vivo observations of PLTs biogenesis in mice that showed shear stress in blood flow is a critical requirement for PLTs shedding from megakaryocytes (MK)^14^, the ex vivo production of PLTs using shear flow-based reactor systems was widely investigated^9,15–18^. These PLTs, however, showed functions inferior to those of bona fide PLTs^15^. One reason was the absence of turbulence, which causes a rapid change in flow directions, in the systems. This finding led to the development of “VerMES”, a reciprocal vertical motion reactor, wherein turbulent flow was kept within an appropriate range of turbulent energy and shear stress, leading to the efficient generation of functional PLTs from imMKCLs in 0.5 L (VerMES0.5) and 10 L (VerMES10) non-disposable glass type tanks^19^.

However, further validations are needed for this system to be clinically practical at larger scales.

Here, we examined whether the same process under good manufacturing practice works with a 50-L single-use (45 L working volume) VerMES tank (VerMES50). In order to obtain intact iPSC-PLTs, the optimal ranges of turbulent energy and shear stress are constant in tanks ranging from 10–50 L. However, one noticeable difference was that the production efficiency, defined by the number of PLTs per imMKCL, decreased with scale. The reason was a larger volume of defective turbulent flow in the bioreactor tank^20^. To resolve this problem, we conducted a mathematical simulation that indicated an additional impeller (three in total) in the reactor is worthwhile. However, to achieve optimal turbulent energy and shear stress, the requisite motion speed against the increased weight of the three impellers in the 50 L tank is not possible with commercial products. Therefore, we designed a novel reactor with a modified rotary mixing impeller and wall structure for easy scale-up.

## Results

### Development of VerMES50 with single-use bag

We previously demonstrated that a 10 L tank (8 L working volume) reciprocal motion-dependent VerMES reactor (VerMES10) is capable of producing 100 billion (10^11) competent iPSC-derived PLTs (iPSC-PLTs)^19^. By applying this production system, we performed the first-in-human clinical trial of iPSC-PLTs transfusions (4 glass tanks, 32 L total working volume; 32 L was required to cover the loss due to two centrifugations for purification and washing)^12,21^ Next, towards industrial scale production, we developed 50 with a good-manufacturing-practice grade, single-use United States Pharmacopoeia standard (USP) class IV polyethylene tank (Supplementary Fig. 1) and with a newly developed motor regulator to produce competent iPSC-PLTs.

**Fig. 1:**
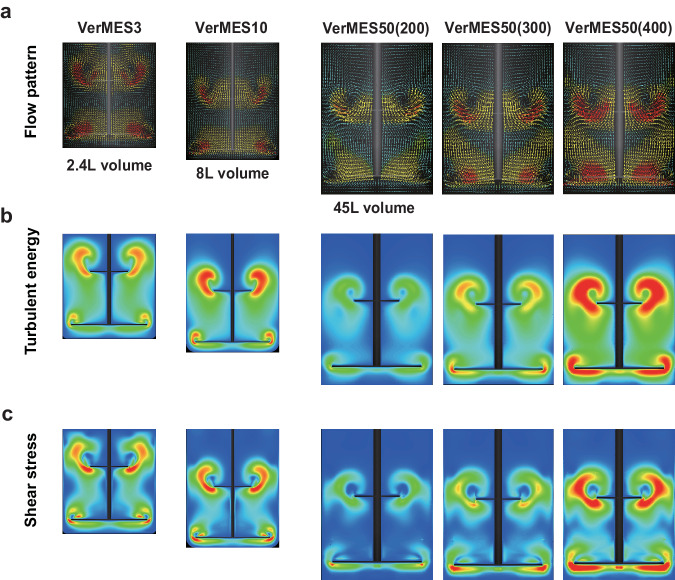
Comparison of VerMES reactor systems. **a** Representative flow patterns of optimized impeller motion speeds in VerMES3 and VerMES10 and three different motion speeds (200, 300, and 400 mm s-1) in VerMES50. Representative results were selected from three-dimensional visualized movies by a computer flow dynamics (CFD) analysis. **b, c** Representative results of turbulent energy (**b**) and shear stress (**c**) for VerMES3, VerMES10, and VerMES50. Data were obtained by synchronizing the flow patterns in **a**.

For VerMES, we previously demonstrated the optimal range of turbulent energy and shear stress are relatively constant up to a 10 L tank^19^. We decided to assess whether the same applies to a 50 L tank by a computational fluidic dynamic (CFD) analysis^19^. In the CFD analysis (Fig. 1a–c and Supplementary Movies 1a–c), 150 mm s-1 motion speed for VerMES10 corresponds to optimal turbulent energy and shear stress^19^ (Table 1) and was used for the aforementioned human clinical trial.

**Table 1:** The CFD simulation of VerMES two-impeller model

Scale	Speed	Turbulent energy	Shear stress	Kolmogorov scale	Shear rate	Vorticity	Dissipation of energy
(L)	(mm s-1)	(m2 s-2)	(Pa)	(μm)	(s-1)	(s-1)	(m2 s-3)
3	150	0.0129	3.93	150	17.29	13.4	0.099
10	150	0.0113	3.29	201	10.53	7.79	0.062
50	200	0.0082	2.38	286	5.60	4.46	0.023
50	300	0.0109	3.18	264	6.67	5.27	0.038
50	400	0.0220	6.41	194	8.64	6.80	0.097

The CFD analysis results show the optimal values of motion speed, turbulent energy, shear stress, Kolmogorov scale (vortex size), shear rate, vorticity, and dissipation of energy for VerMES3, VerMES10, and VerMES50.

To determine the motion speed in VerMES50 for equivalent optimal turbulent energy and shear stress, we compared the real speed of the impeller movement between VerMES10 and VerMES50. Because of the heavier impellers in VerMES50, the motor regulation system in the two reactors is different, and the real speed of the impeller in VerMES50 was less and plateaued earlier (Supplementary Fig. 2). With this point in mind, we used the CFD simulation to determine that 200, 300, and 400 mm s-1 reciprocal motion speeds in VerMES50 corresponded to turbulent energies of 0.0082, 0.0109, and 0.0220 m2 s-2, respectively (Fig. 1b and Table 1). Therefore, 300 mm s-1 was selected as the optimal speed. The CFD analysis also revealed that 300 mm s-1 is optimal for shear stress in VerMES50 (Fig. 1c and Table 1).

### iPSC-PLTs production in VerMES50

**Fig. 2:**
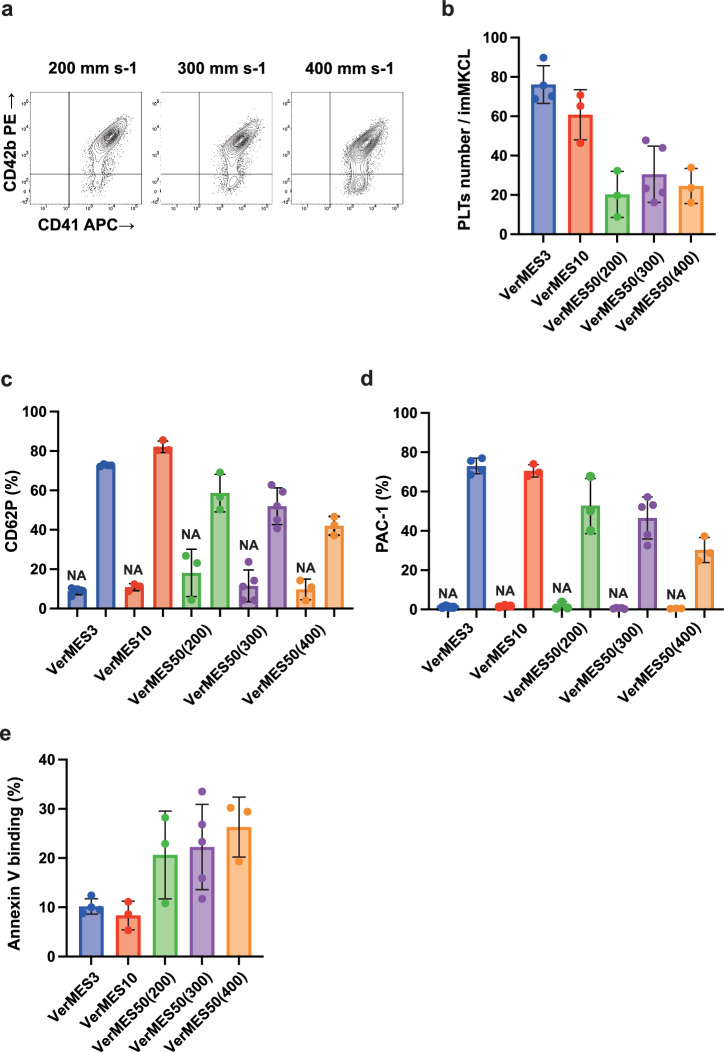
Relationship between motion speed and iPSC-PLTs yield. **a** Representative flow cytometry dot plots of CD41 (X-axis) and CD42b (GPIbα) (Y-axis) from VerMES50 set at 200, 300, and 400 mm s-1. **b** Production of CD41^+^CD42b^+^ iPSC-PLTs from imMKCLs was evaluated in VerMES3: Blue bar (*N* = 4), VerMES10: Red bar (*N* = 3), and VerMES50 at 200: Green bar (*N* = 3), 300: Purple bar (*N* = 5), or 400 mm s-1: Orange bar (*N* = 3). Values were taken at Dox-OFF day 6 (mean ± SD). **c–e** CD62P (P-selectin) (**c**), PAC-1 binding (**d**), and Annexin V binding (**e**) were quantified by flow cytometry. CD62P expression and PAC-1 binding were measured before and after stimulation with 40 μM TRAP and 100 μM ADP. Annexin V binding was evaluated in the presence or absence of 20 μM ionomycin. PLTs platelets, imMKCL immortalized megakaryocyte cell line.

We used imMKCLs clone 7, which we have extensively evaluated previously for iPSC-PLTs production^13,19,22^. The production efficiency was evaluated by the CD41^+^CD42b^+^ PLTs number at day 6 of the DOX-OFF stage per imMKCL (number at DOX-OFF day 0 for MK maturation) (Fig. 2a)^13,19,22^. Figure 2b shows less efficiency in VerMES50 at all three speeds compared to VerMES3 or VerMES10. Of those three speeds, 300 mm s-1 had the best iPSC-PLTs production (Fig. 2b). Notably, two trials at 300 mm s-1 resulted in the highest production (52 and 48 PLTs per imMKCL). CD62P (P-selectin) expression and PAC-1 binding, which reflects the activated form of integrin αIIbβ3 required for PLTs aggregation, were also evaluated. The observations indicated that VerMES50 produces iPSC-PLTs of lower quality than VerMES3 or VerMES10 (Fig. 2c, d). Annexin V binding, which reflects phosphatidyl serine expression on the cell membrane, also indicated poorer quality (Fig. 2e).

### In vivo function of iPSC-PLTs from VerMES50

**Fig. 3:**
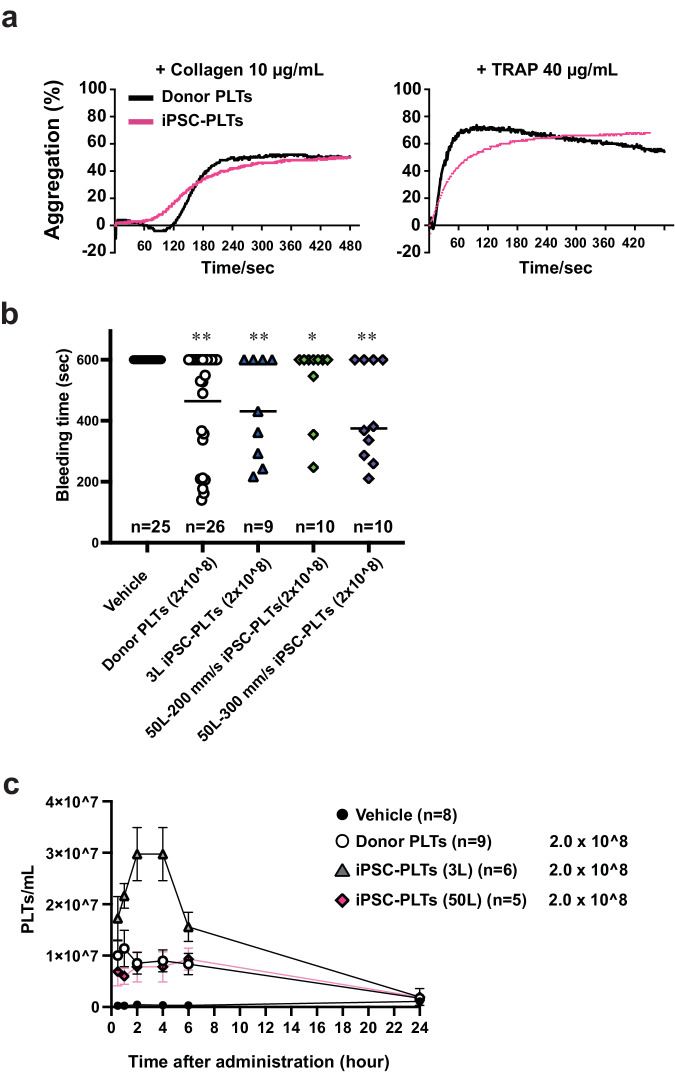
Functional assessment in vitro and in vivo of VerMES50 iPSC-PLTs. Evaluations were done after the depletion of remaining imMKCLs and subsequent washing of iPSC-PLTs. **a** Aggregation of donor PLTs or iPSC-PLTs post agonist stimulation (10 μg/mL collagen or 40 μg/mL TRAP) based on light transmission. Donor PLTs: black line, VerMES50 iPSC-PLTs: pink line. **b** Bleeding times after transfusion were measured in a thrombocytopenia mouse model. Vehicle (bicarbonated Ringer’s solution with 10% ACD-A and 2.5% albumin, black dots), donor PLTs (white dots), and iPSC-PLTs (3 L: blue triangle, 50L-200 mm s-1: green diamonds, 50L-300 mm s-1: purple diamonds) were transfused at a dose of 2 × 10^8 per mouse. The tail puncture and measurements of bleeding time were performed 2 h after the transfusion. Lines show medians. *P < 0.05, **P < 0.01, Mann-Whitney test vs. vehicle. **c** Post transfusion kinetics in the thrombocytopenia mouse model. Vehicle (bicarbonated Ringer’s solution with 10% ACD-A and 2.5% albumin, black dots), donor PLTs (white dots), and iPSC-PLTs (3 L: grey triangles, 50 L: pink diamonds) were transfused at a dose of 2 × 10^8 per mouse. Blood sampling was done 0.5, 1, 2, 4, 6, and 24 h after the transfusion. PLTs platelets, iPSC-PLTs induced-pluripotent-stem-cell-derived platelets.

We next tested the in vitro aggregation and in vivo functions (bleeding time and circulation kinetics post-transfusion) of the iPSC-PLTs. First, transmission light microscopy was used to compare the aggregation of donor PLTs and iPSC-PLTs generated at 300 mm s-1 in VerMES50. When stimulated by 10 μg/mL collagen or 40 μM thrombin receptor activating peptide 6 (TRAP 6), the maximum aggregations were similar (Fig. 3a).

Next, we assessed the in vivo hemostatic function of iPSC-PLTs generated at 200 or 300 mm s-1. These iPSC-PLTs were transfused into thrombocytopenic mice, and bleeding times were compared to vehicle treatment (Fig. 3b and Supplementary Fig. 3). As positive controls, donor human PLTs or iPSC-PLTs from VerMES3 were administered (2 × 10^8^ PLTs per mouse). No bleeding events were terminated in the vehicle group within the detection time limit of 600 s (*n* = 25), but hemostatic action was observed when PLTs were administered. Tail bleeding stopped in 60% of mice (6 of 10 mice) at a dose of 2 × 10^8^ PLTs per mouse and in 86% (7 of 8 mice) at 6 × 10^8^ PLTs per mouse for iPSC-PLTs from VerMES50 at 300 mm s-1. Moreover, the hemostatic function appeared to be dose-dependent (median: 374.5 s at 2 × 10^8^ PLTs, 276.5 s at 6 × 10^8^ PLTs) (Fig. 3b). iPSC-PLTs generated at 200 mm s-1 showed less hemostatic function and stopped bleeding in only 30% of mice (3 of 10 mice) administered 2 × 10^8^ PLTs (Fig. 3b and Supplementary Fig. 3).

**Fig. 4:**
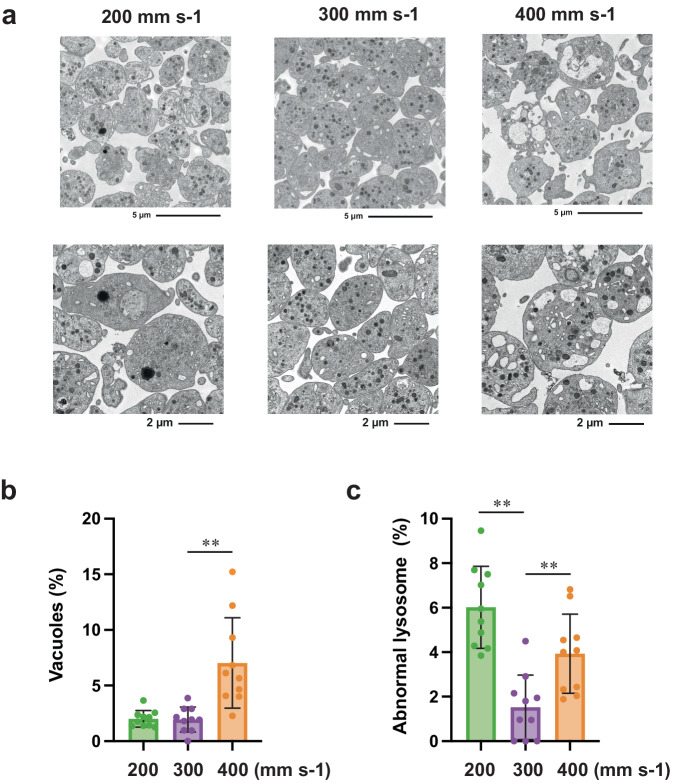
Morphological evaluation of VerMES50 iPSC-PLTs. **a** TEM images of iPSC-PLTs from VerMES50 set at three speeds. Scale bars: 5 μm (upper panels) and 2 μm (lower panels). **b** Percentage of iPSC-PLTs positive for vacuoles. VerMES50 at 200: Green bar (*N* = 10), 300: Purple bar (*N* = 10), or 400 mm s-1: Orange bar (*N* = 10). **P < 0.01, Mann-Whitney test vs. 300 mm s-1. **c** Percentage of iPSC-PLTs positive for abnormal lysosomes. Evaluation methods are described in Methods. **P < 0.01, Mann-Whitney test vs. 300 mm s-1.

Next, we examined the circulation kinetics of transfused iPSC-PLTs into a thrombocytopenic mouse (treated with irradiation and anti-mouse CD42b (GPIbα) antibody infusion). iPSC-PLTs from VerMES3 (2 × 10^8^ per mouse) showed increased human CD41^+^ count until 4 h after the infusion, whereas iPSC-PLTs from VerMES50 (300 mm s-1; 2 × 10^8^ per mouse) displayed less yield (Fig. 3c). Overall iPSC-PLTs from VerMES50 at all three speeds demonstrated poorer function than iPSC-PLTs from VerMES3 or VerMES10 (Fig. 2). Moreover, among iPSC-PLTs from VerMES50, only those at 300 mm s-1 showed an intact structure by transmission electron microscopy (TEM) examination (Fig. 4a). VerMES50 iPSC-PLTs manufactured at other speeds showed impaired open canalicular systems based on the number of vacuoles (400 mm s-1 in Fig. 4b) or increased area of abnormal lysosomes (200 or 400 mm s-1 in Fig. 4c).

### Importance of optimal turbulence in VerMES

**Fig. 5:**
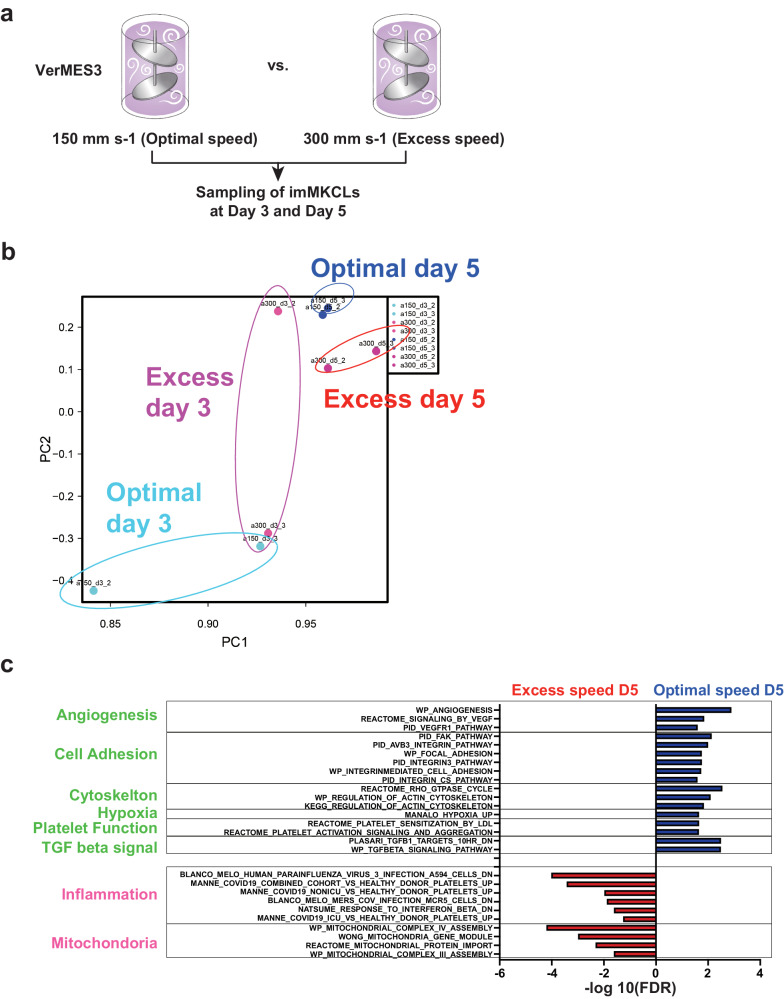
Excessive shear stress and turbulent energy upregulate the expression of immune- and mitochondria-related genes and downregulate the expression of PLTs function-related genes. **a** Cells in VerMES3 were compared on days 3 and 5. **b** A principal component analysis of the relationship between optimal speed (Opt) and excess speed (Exc) with shear stress and turbulent energy. **c** Bar graphs showing enriched pathways in the Opt and Exc groups on day 5. imMKCLs immortalized megakaryocyte cell line, FDR false discovery rate.

**Fig. 6:**
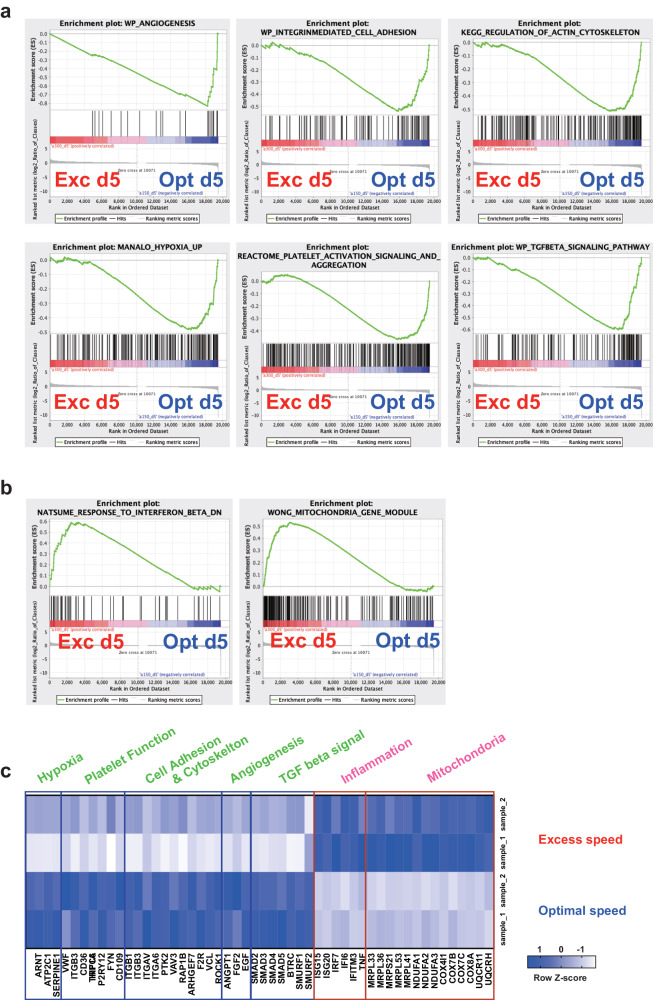
Differential analysis of PLTs production conditions using gene set enrichment analysis. **a,** **b** A gene set enrichment analysis shows the increased expression of gene sets in the optimal speed (Opt) and excess speed (Exc) groups. **c** A heatmap of differentially expressed genes in the Opt and Exc groups on day 5.

To address the reason for the abnormalities and poor function, RNA sequencing was performed on days 3 and 5 of the Dox-OFF stage. Day 5 was chosen over day 6, because most PLTs production was observed during days 5–6, and day 3 was chosen because the RNA preparation was unaffected by the speed on that day (Fig. 5a). A principal component analysis (PCA) showed significantly separate clustering at day 5 but not on day 3 (Fig. 5b), indicating that the final 2-3 days of the total 6-day culture is critical, wherein the demarcation membrane system (DMS) and proplatelets are observed^19^. A pathway enrichment analysis of day-5 cells showed upregulated gene sets for angiogenesis, cell adhesion, cytoskeleton, hypoxia, PLTs function, and TGF beta signaling at 150 mm s-1 inVerMES3 (which corresponds to 300 mm s-1 in VerMES50) (Fig. 5c), and upregulated inflammation- and mitochondria-related genes at 300 mm s-1 VerMES3 (which corresponds to 400 mm s-1 in VerMES50) (Fig. 5c). These results were confirmed by a gene set enrichment analysis (Fig. 6a, b). Among those gene sets, representative gene lists are depicted as individual gene groups (Fig. 6c). In VerMES3 imMKCLs at 150 mm s-1, HIF1A (hypoxia-inducible factor 1 alpha)^23,24^, ARNT (aryl hydrocarbon receptor nuclear translocator, or HIF1B)^24^, and TGFβ^25^ signaling, which are all required for MK maturation, and vWF, integrins, FYN, PTK2 (FAK), and RAP1B, which are required for PLTs function and thrombopoiesis^26^, were upregulated. In contrast, inflammation-related genes, including ISG15, IFITM3^27,28^, and TNF, and dysregulated mitochondria-related genes, such as the MRPL family^29^, were upregulated at 300 mm s-1 (Fig. 6c). Abnormal mitochondria-related activation is known to induce apoptosis^30,31^, which may explain why VerMES50 iPSC-PLTs at 400 mm s-1, which causes excessive turbulent energy and shear stress, are of poorer quality and show abnormal structures.

### Larger defective turbulent flow space is associated with reduced production efficiency in VerMES50

**Fig. 7:**
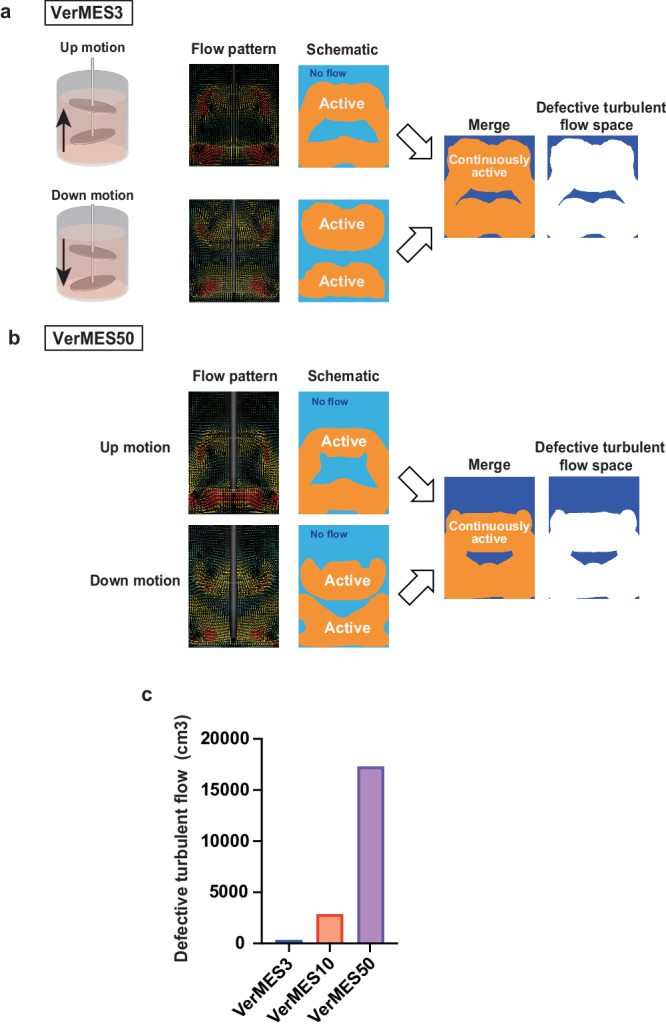
Detection of defective turbulent flow space in VerMES reactor by CFD simulation. **a** Representative flow patterns of VerMES3 showing different positions of impellers (up motion and down motion). The orange-colored area represents active turbulent flow space in both (up and down) positions. There is no turbulence in the light-blue colored area, which represents the defective turbulent flow space. The dark blue area represents defective turbulent flow space in the merged view. **b** Representative flow patterns of VerMES50. **c** The volume of defective turbulent flow space in VerMES3: Blue bar, VerMES10: Red bar, and VerMES50 at 300 mm s-1: Purple bar motion speed based on a CFD analysis.

To explain why the production efficiency in VerMES50 was less than in VerMES3 or VerMES10, we estimated the volume of defective turbulent flow space using the sequential three-dimensional flow pattern results from the CFD simulation, which depended on the impeller position. As shown in Fig. 7a, the flow pattern of the up and down motions caused different turbulent flow. Merging the flow patterns revealed different defective turbulent flow spaces in VerMES3 and VerMES50 (Fig. 7a, b). Moreover, the size of the space grew with the size of the reactor volume (Fig. 7c).

### Putative CFD suggests a strategy for large-scale manufacturing

**Fig. 8:**
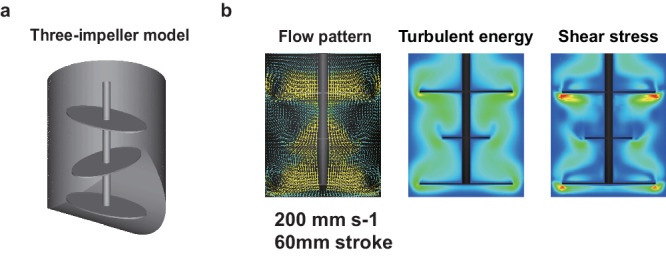
Three-impeller model by CFD simulation in putative VerMES50 tank. **a** Illustration of three impellers of a putatively improved VerMES50. **b** Flow pattern, turbulent energy results, and shear stress at 200 mm s-1 motion speed with 60 mm strokes.

The PLTs yields per imMKCL in VerMES50 was significantly less than those of VerMES3 or VerMES10 (Fig. 2b). We attributed the poorer production efficiency to a larger defective turbulent flow space in VerMES50 (Fig. 7c) and speculated that the short strokes by the two impellers, particularly observed in the upper space of the VerMES50 tank, are important factors (Fig. 7b, right). We conducted an additional CFD simulation using three impellers (Fig. 8a and Supplementary Movie 2). The simulation showed an improved flow pattern with less defective turbulent flow space (Fig. 8b). With three impellers, optimal turbulent energy, and shear stress can be achieved at 200 mm s-1 motion speed in VerMES50 compared with two impellers if using the same stroke distance (60 mm; Fig. 8b and Table 2).

**Table 2:** The CFD simulation of VerMES50 Three-impeller model

Scale	Speed	Turbulent energy	Shear stress	Kolmogorov scale	Shear rate	Vorticity	Dissipation of energy
(L)	(mm s-1)	(m2 s-2)	(Pa)	(μm)	(s-1)	(s-1)	(m2 s-3)
50	200	0.0112	3.35	198	6.22	4.32	0.0279

The CFD analysis indicated that three impellers give optimal motion speed, turbulent energy, shear stress, Kolmogorov scale, shear rate, vorticity, and dissipation of energy.

The reciprocal movement in the two-impeller model results in VerMES50 having a faster optimal speed than VerMES10 (setting speeds of 300 mm s-1 vs. 150 mm s-1, real average speeds of 130.4 mm s-1 vs. 100.7 mm s-1). An additional third impeller increases the weight of the motor regulator, thus requiring more power for reciprocal motion. However, such a motor regulator is currently unavailable commercially. Therefore, a rotary reactor may be preferred for large-scale manufacturing. However, while commercial rotary reactors can exceed 1000 L, they do not provide optimal turbulence in a uniform distribution^19^.

Based on the flow pattern analysis, we developed a novel reactor system, which we call HS606SB, by modifying the rotary-type impeller to have a long shaft, and the tank to enable a uniform cell distribution (Supplementary Fig. 4a). A CFD analysis showed a reciprocal motion-like flow pattern controlled by the rotation speed, whereby 200 rpm in HS606SB-3L or 140 rpm in HS606SB-10L achieved flow patterns resembling those of VerMES3 or VerMES10, respectively (Supplementary Fig. 4b).

HS606SB-3L generated 75 CD41^+^CD42b^+^ iPSC-PLTs per imMKCL cell (Supplementary Fig. 4c), 10% of which showed annexin V binding (Supplementary Fig. 4d). Transmission electron microscopy revealed intact structures (Supplementary Fig. 4f), and good in vivo bleeding times post-transfusion (Supplementary Fig. 4e) demonstrated the feasibility of HS606SB-3L. Therefore, HS606SB-3L may be the basis for future large-scale manufacturing of iPSC-PLTs.

## Discussion

The limited supply of PLTs for PLTs transfusions has led to intensive research on ex vivo donor independent PLTs products. iPSCs are one attractive source, especially since they have been used to produce imMKCLs, which shed PLTs of good function^13^. However, the PLTs yield from imMKCLs is relatively poor compared to in vivo circumstances^32–35^. We have shown that controlling for turbulent energy and shear stress in bioreactors can significantly increase the yield^19^. In the present study, we show the best values for these two physical factors in 50 L bioreactors (45 L volume), which is 5.6 times larger than any previously tested volume (maximum 8 L in 10 L tank), a substantial scaling-up step towards the affordable generation of clinical-grade iPSC-PLTs^19^.

To manufacture PLTs products in reactors less than 1 L, two approaches have been considered. One is to improve the original flow chamber using unique materials, such as silkworms with extracellular matrix proteins of optimal stiffness^16,36^. The other is to directly apply physical stimulation (i.e., continuous shear stress with membrane filtration) to enable fragmentation of the MK cytoplasm, but most attempts have failed to produce an adequate number of intact and fully functional PLTs, i.e., more than 100 billion^37–39^. Therefore, we previously evaluated the behavior of MKs in mouse skull bone marrow to elucidate the effects of turbulence on PLTs biogenesis^19^. The same study showed the usefulness of the VerMES reactor system, where optimal levels of turbulent energy and shear stress contributed to the PLTs yield and quality. iPSC-PLTs made from VerMES reactors of multiple volumes (0.5 L, 3 L, and 10 L) were functionally comparable with donor PLTs^19^. Based on this success, we commenced the first-in-human clinical trial of iPSC-PLTs produced using four VerMES10 reactors (total 32 L volume)^12^. For future clinical and commercialization purposes, in the present study, we tested the feasibility of the VerMES50 reactor for the production, finding a motor regulator system with three impellers is preferred. Furthermore, we found evidence that not only turbulent energy and shear stress but also the volume of defective turbulent flow space are crucial factors for the production (Tables 1, 2 and Figs. 7, 8).

Turbulent energy and shear stress are dependent on the stirring or motion speed inside the bioreactor. In VerMES50 with two impellers, we found at speeds too low, the yield was poor (200 mm s-1, Fig. 2b), and at speeds too high (400 mm s-1), the cells had poor function (Fig. 2b–e and Supplementary Fig. 3). Further analysis revealed that the poor function was correlated with an increase in the expression of inflammation- and mitochondrial-associated genes. On the other hand, at speeds inducing optimal turbulence, the expression of genes for MK maturation and thrombopoiesis were upregulated. Meanwhile, RNA sequencing indicated that excessive shear stress in the manufacturing process induces the expression of inflammation genes commonly observed in aging cells^40^ and mitochondrial genes^29^ associated with PLTs hyperactivity^41^ or immune-skewed MKs^42^ (Figs. 5, 6).

However, VerMES50 showed a defective turbulent flow space (Fig. 7), suggesting that its two-impeller design is not appropriate for the large-scale manufacturing of iPSC-PLTs. CFD simulations indicated the benefit of a three-impeller design (Fig. 8a, b) for improving the production efficiency. Therefore, here we propose a new rotary-type reactor.

In the commercialized manufacturing of antibody-producing cell lines, rotary reactors can operate at volumes over 1000 L. Noting the importance of homogeneous cell distribution in the conventional rotary reactor (i.e., XDR-10 or XDR-50)^19^, by modifying the rotary impeller, we designed a new rotary reactor, HS606SB, for future PLTs manufacturing (Supplementary Fig. 4a). Although the volume tested for PLTs function was limited to 3 L, compared to VerMES3, this new design has promise for scaling above 50 L since it does not share the same design limitations (i.e., new motor regulator) as VerMES50. However, even HS606SB will need modifications to its impeller and tank to reduce the defective turbulent flow space at higher volumes (Supplementary Fig. 4b and Supplementary Table 1).

Taken together, PLTs manufacturing at larger scales will need not only to consider turbulent energy and shear stress but also the defective turbulent flow space in the tank.

## Methods

### Cell preparation and culture

#### Dox-ON proliferation culture

The expansion culture for imMKCLs clone 7 (Dox-ON stage) was performed as previously reported^19^. The cell culture was expanded by sequential upsizing starting from 10-cm dishes under static state, to 125 mL and 500 mL Corning^®^ Erlenmeyer cell culture flasks (Sigma-Aldrich, #CLS431143) under shaking using a Lab-Therm shaker (Kuhner), and finally, 10 L- and 50 L-scale CELLBAG^TM^ operated by ReadyToProcess WAVE™ 25 (Cytiva, #CB0010L10-03 and #CB0050L10-02) with rocking. The cell concentrations were between 1 × 10^5^ and 2 × 10^6^ cells/mL throughout the culture.

#### Dox-OFF differentiation culture

For PLTs production after imMKCLs maturation (Dox-OFF stage)^12,19^, approximately 2–4 × 10^5^ cells/mL were mixed for 6 days in a reactor tank.

### VerMES reactor

All VerMES reactors used were constructed by SATAKE MultiMix Corporation (Toda, Saitama, Japan). The motion speeds used for VerMES50 were 200, 300, and 400 mm s-1, the stroke size was 60 mm, and the working volume was 45 L (50 L tank). The working volumes of VerMES0.5, VerMES3, and VerMES10 were 0.3 L, 2.4 L and 8 L, respectively, and used as controls^19^.

### HS606SB reactor

The HS606SB reactor was constructed by SATAKE MultiMix Corporation. The rotation speed was 200 rpm (3 L tank) or 140 rpm (10 L tank), and the working volumes were 2.4 L (3 L tank) and 8 L (10 L tank).

### Physical flow simulations in bioreactors

Simulations of single-phase flows and solid-liquid multiphase flows were performed using CFD software that included the thermal fluid analysis module of Fluent 2022 R1 (ANSYS, Inc.). To ensure high accuracy of the simulation results, we conducted validations using PTV (Particle Tracking Method). To verify the turbulence model, we performed the Realizable *k*-ε model^43^. The following parameters were calculated using the equations and the results are summarized in Tables 1, 2, and Supplementary Table 2.

**An unsteady analysis was performed as follows:**

**[Solver]**

Solver type: Pressure-based solver

Time: Steady (HS606SB)

Transient (VerMES)

Solution method: SIMPLE method

**[Turbulence model]**

Realizable *k*-ε model

**[Near-wall treatment approach]**

Menter-Lechner

**[Boundary conditions]**

Liquid surface: Slip wall

Other wall: No slip wall

**[Fluid]**

Fluid: incompressible fluid.

Fluid density: *ρ* = 1005 kg m3

Fluid viscosity: *µ* = 3 mPa·s

**[Mesh]**

The parameters of each bioreactor are defined in Supplementary Table 2.

**[Equations]**

*μ*: viscosity coefficient

*ν*: kinematic viscosity

*κ*: turbulent energy

*ε*: energy dissipation

*γ*: shear rate

*τ*: shear stress

*ω*: vorticity

*λ*: Kolmogorov scale

*Turbulent energy, κ*

1 $$\frac{\partial \left(\rho k\right)}{\partial t}+\frac{\partial \left(\rho \bar{{U}_{i}}k\right)}{\partial {x}_{i}}=\frac{\partial }{\partial {x}_{i}}\left\{\left(\mu +\frac{{\mu }_{t}}{{\sigma }_{k}}\right)\frac{\partial k}{\partial {x}_{i}}\right\}+{P}_{k}-\rho \varepsilon$$

*Energy dissipation of turbulence*

2 $$\frac{\partial \left(\rho \varepsilon \right)}{\partial t}+\frac{\partial \left(\rho \bar{{U}_{i}}\varepsilon \right)}{\partial {x}_{i}}=\frac{\partial }{\partial {x}_{i}}\left\{\left(\mu +\frac{{\mu }_{t}}{{\sigma }_{\varepsilon }}\right)\frac{\partial \varepsilon }{\partial {x}_{i}}\right\}+\rho {C}_{1}{S}_{\varepsilon }-\rho {C}_{2}\frac{{\varepsilon }^{2}}{k+\sqrt{\mu \varepsilon }}$$

Turbulent viscosity coefficient

3 $${\mu }_{t}={C}_{\mu }\rho \frac{{k}^{2}}{\varepsilon }$$

4 $${{{{{\rm{Vector}}}}}}\;\;\;{{{{{\bf{v}}}}}}=\left(u,v,w\right)$$

5 $${{{{{\rm{Shear\; stress}}}}}}\;\;\;\;\tau =\mu \dot{\gamma }$$

Strain rate

6 $$\dot{\gamma }=\sqrt{2\left[{\left(\frac{\partial u}{\partial x}\right)}^{2}+{\left(\frac{\partial v}{\partial y}\right)}^{2}+{\left(\frac{\partial w}{\partial z}\right)}^{2}\right]+{\left(\frac{\partial u}{\partial y}+\frac{\partial v}{\partial x}\right)}^{2}+{\left(\frac{\partial v}{\partial z}+\frac{\partial w}{\partial y}\right)}^{2}+{\left(\frac{\partial w}{\partial x}+\frac{\partial u}{\partial z}\right)}^{2}}$$

Vorticity

7 $$\omega 	={{{{{\rm{||rot}}}}}}\; {{{{{\bf{v||}}}}}}\\ 	=\sqrt{{\left(\frac{\partial w}{\partial y}-\frac{\partial v}{\partial z}\right)}^{2}+{\left(\frac{\partial u}{\partial z}-\frac{\partial w}{\partial x}\right)}^{2}+{\left(\frac{\partial v}{\partial x}-\frac{\partial u}{\partial y}\right)}^{2}}$$

Kolmogorov scale

8 $$\lambda ={\left(\frac{{\left(\mu /\rho \right)}^{3}}{\varepsilon }\right)}^{\frac{1}{4}}$$

### RNA sequencing

RNA was extracted using the RNeasy Micro Plus Kit (Qiagen/#74034) from cultured imMKCLs at DOX-OFF day 3 and day 5. For each sample, total RNA (maximum 10 ng) was processed using the SMART-Seq v4 Ultra Low Input RNA Kit for Sequencing (Clontech/#634890). cDNA was fragmented using an S220 Focused-ultrasonicator (Covaris). The cDNA library was then amplified using a NEBNext® Ultra™ DNA Library Prep Kit for Illumina (cat. #E7370L, New England Biolabs). The NEBnext library size was estimated using a bioanalyzer with an Agilent High Sensitivity DNA Kit. Sequencing was performed using a HiSeq 2500 (Illumina) with a single-read sequencing length of 60 bp.

TopHat (version 2.1.1; with default parameters) was used to map to the reference genome (UCSC/hg19) with annotation data from iGenomes (Illumina). Gene expression levels were quantified using Cuffdiff (Cufflinks version 2.2.1; with default parameters).

### Platelets purification, washing, and concentration

After 6 days of Dox-OFF, all medium containing iPSC-PLTs and residual imMKCLs were concentrated and washed using a hollow fiber filtration and an ACP centrifugation system (HAEMONETICS ACP^®^215)^19,21^. PLTs were re-suspended in PLTs storage solution consisting of bicarbonated Ringer’s solution with ACD-A solution (10%) plus 2.5% albumin^44^.

### Flow cytometry analysis

iPSC-PLTs at Dox-OFF day 6 were used in the functional study. A flow cytometry analysis was performed using FACSVerse. The following antibodies were used: anti-hCD41-APC (#303710, BioLegend Inc), anti-hCD42b-PE (#303906, BioLegend Inc), anti-hCD62P-Brilliant Violet 421 (#304910, BioLegend Inc), FITC-PAC-1 (#340507, BD Biosciences), and FITC Annexin V (#556419, BD Biosciences). The number of PLTs was determined using Trucount Tubes (BD Biosciences, #340334). The number of PLTs per imMKCL was defined as the final count of CD41^+^CD42b^+^ iPSC-PLTs at Dox-OFF day 6 divided by the number of imMKCLs cells at Dox-OFF day 0. Before and after activation by 40 µM TRAP-6 (BACHEM #H-8365.0005) and 100 µM ADP (Sigma Aldrich #A-2754) or 20 µM ionomycin (Wako #091-05833), PLTs were stained for PAC1, CD62P and Annexin V. Gating strategy refers to Supplement Fig. 5.

### Functional study

PLTs aggregation was done as previously described^19,21^. PLTs were prepared to 3.0 × 10^8^ PLTs/mL, and 200 μL of the preparation was measured for light transmission using an aggregometer (MCM Hematracer 313 M, Model PAM-12 C; LMS, Japan) in the presence of agonists (10 μg/mL collagen, or 40 μM TRAP) for 8 min at 37 °C.

For the in vivo analysis, the immune-deficient mouse strain NOG (Central Institute for Experimental Animals, Kawasaki, Kanagawa, Japan) or NSG-SGM3 (Charles River Laboratories Japan, Yokohama, Japan) was used. All animal experiments at Kyoto University conformed to ethical principles and guidelines approved by the Ethical Committee of Kyoto University. Thrombocytopenia was induced by 2.4 Gy gamma irradiation to 8–9-week-old male NOG mice. Nine days after the irradiation, the mice were infused with anti-mouse CD42b antibody (emfret Analytics#R300) at a dose of 50 ng/gram mouse body weight to deplete the PLTs, followed by a transfusion of 2 × 10^8^ PLTs (100 μL of 2 × 10^9^ PLTs/mL) or vehicle to the tail vein while the mice were awake. Then, hemostasis was assessed by tail bleeding, which was caused by a puncture with a 23 G needle to the tail artery 2 cm from the tip. The punctured tail was immersed with warmed saline (37 °C), and bleeding time was counted up to 600 s until the outflow of blood was discontinued. For the circulation study, the mice were prepared as above, and peripheral blood sampling was done from the external jugular vein before and 0.5, 1, 2, 4, 6, and 24 h after the vehicle or PLTs transfusion. Blood samples were fixed with ThromboFix (BECKMAN COULTER/#6607130), and the number of PLTs was counted by flow cytometry.

### Electron microscopy

Specimens were prepared and detected with a transmission electron microscope (HT-7700; Hitachi, Tokyo, Japan) operating at 80 kV as previously described^18^. To quantify the abnormal structure, ten transmission electron microscopy pictures were randomly selected, and all iPSC-PLTs of individual pictures were counted and classified into three different groups: normal structure, presence of two vacuoles of large size, and abnormal lysosomes with large black spots. The percentage of iPSC-PLTs positive for vacuoles or abnormal lysosomes was measured.

### Statistical analysis

Statistical analysis was performed for the hemostatic function study. Each group of data was subjected to an unpaired two-tailed Mann-Whitney test.

### Reporting summary

Further information on research design is available in the Nature Portfolio Reporting Summary linked to this article.

### Supplementary information

Supplementary Information

Description of Additional Supplementary Files

supplemental movie 1a

supplemental movie 1b

supplemental movie 1c

supplemental movie 2

Reporting Summary

## Data Availability

Raw RNA-seq data are available in the GEO repository under accession code GSE262455. The other data supporting the findings of this study are available from the corresponding author upon reasonable request.

## References

[1] The United States Department of Health and Human Services. The 2011 National Blood Collection and Utilization Survey Report. https://wayback.archive-it.org/3922/20190926121044/https:/www.hhs.gov/sites/default/files/ash/bloodsafety/2011-nbcus.pdf (2011).

[2] Estcourt LJ (2014). Why has demand for platelet components increased? A review. Transfus Med..

[3] Dolgin E (2017). Bioengineering: Doing without donors. Nature.

[4] Ngo A, Masel D, Cahill C, Blumberg N, Refaai MA (2020). Blood Banking and Transfusion Medicine Challenges During the COVID-19 Pandemic. Clin. Lab Med..

[5] Stanworth SJ (2020). Effects of the COVID-19 pandemic on supply and use of blood for transfusion. Lancet Haematol.

[6] Wiita AP, Nambiar A (2012). Longitudinal management with crossmatch-compatible platelets for refractory patients: alloimmunization, response to transfusion, and clinical outcomes (CME). Transfusion.

[7] Estcourt LJ (2017). Guidelines for the use of platelet transfusions. Br. J. Haematol..

[8] Takayama N (2010). Transient activation of c-MYC expression is critical for efficient platelet generation from human induced pluripotent stem cells. J. Exp. Med..

[9] Thon JN (2014). Platelet bioreactor-on-a-chip. Blood.

[10] Moreau T (2016). Large-scale production of megakaryocytes from human pluripotent stem cells by chemically defined forward programming. Nat. Commun..

[11] Sim X, Poncz M, Gadue P, French DL (2016). Understanding platelet generation from megakaryocytes: implications for in vitro-derived platelets. Blood.

[12] Sugimoto N (2022). iPLAT1: the first-in-human clinical trial of iPSC-derived platelets as a phase 1 autologous transfusion study. Blood.

[13] Nakamura S (2014). Expandable megakaryocyte cell lines enable clinically applicable generation of platelets from human induced pluripotent stem cells. Cell Stem Cell.

[14] Junt T (2007). Dynamic visualization of thrombopoiesis within bone marrow. Science.

[15] Nakagawa Y (2013). Two differential flows in a bioreactor promoted platelet generation from human pluripotent stem cell-derived megakaryocytes. Exp. Hematol..

[16] Di Buduo CA (2015). Programmable 3D silk bone marrow niche for platelet generation ex vivo and modeling of megakaryopoiesis pathologies. Blood.

[17] Blin A (2016). Microfluidic model of the platelet-generating organ: beyond bone marrow biomimetics. Sci. Rep..

[18] Martinez AF, McMahon RD, Horner M, Miller WM (2017). A uniform-shear rate microfluidic bioreactor for real-time study of proplatelet formation and rapidly-released platelets. Biotechnol. Prog..

[19] Ito Y (2018). Turbulence Activates Platelet Biogenesis to Enable Clinical Scale Ex Vivo Production. Cell.

[20] Gaul, L. & Stein, E. *The History of Theoretical, Material and Computational Mechanics - Mathematics Meets Mechanics and Engineering* (ed Erwin Stein), 385–398. (Springer Berlin Heidelberg, 2014).

[21] Sugimoto N (2022). Production and nonclinical evaluation of an autologous iPSC-derived platelet product for the iPLAT1 clinical trial. Blood adv.

[22] Sone M (2021). Silencing of p53 and CDKN1A establishes sustainable immortalized megakaryocyte progenitor cells from human iPSCs. Stem Cell Rep..

[23] Qi J (2017). Downregulation of hypoxia-inducible factor-1α contributes to impaired megakaryopoiesis in immune thrombocytopenia. Thromb. Haemost..

[24] Nguyen-Khac F (2006). Functional analyses of the TEL-ARNT fusion protein underscores a role for oxygen tension in hematopoietic cellular differentiation. Oncogene.

[25] Badalucco S (2013). Involvement of TGFbeta1 in autocrine regulation of proplatelet formation in healthy subjects and patients with primary myelofibrosis. Haematologica.

[26] Stefanini L (2018). Functional redundancy between RAP1 isoforms in murine platelet production and function. Blood.

[27] Perng YC, Lenschow DJ (2018). ISG15 in antiviral immunity and beyond. Nat. Rev. Microbiol..

[28] Campbell RA (2019). Human megakaryocytes possess intrinsic antiviral immunity through regulated induction of IFITM3. Blood.

[29] Huang, G., Li, H. & Zhang, H. Abnormal Expression of Mitochondrial Ribosomal Proteins and Their Encoding Genes with Cell Apoptosis and Diseases. *Int. J. Mol. Sci.* **21**10.3390/ijms21228879 (2020). 10.3390/ijms21228879 PMC7700125 33238645

[30] Zharikov S, Shiva S (2013). Platelet mitochondrial function: from regulation of thrombosis to biomarker of disease. Biochem. Soc. Trans..

[31] Leytin V (2004). Pathologic high shear stress induces apoptosis events in human platelets. Biochem. Biophys. Res. Commun..

[32] Zhang L (2012). A novel role of sphingosine 1-phosphate receptor S1pr1 in mouse thrombopoiesis. J. Exp. Med..

[33] Nishimura S (2015). IL-1α induces thrombopoiesis through megakaryocyte rupture in response to acute platelet needs. J. Cell Biol..

[34] Lefrançais E (2017). The lung is a site of platelet biogenesis and a reservoir for haematopoietic progenitors. Nature.

[35] Potts, K. S. et al. Membrane budding is a major mechanism of in vivo platelet biogenesis. *J. Exp. Med*. **217**10.1084/jem.20191206 (2020). 10.1084/jem.20191206 PMC7478734 32706855

[36] Pallotta I, Lovett M, Kaplan DL, Balduini A (2011). Three-dimensional system for the in vitro study of megakaryocytes and functional platelet production using silk-based vascular tubes. Tissue Eng. Part C Methods.

[37] Lasky LC, Sullenbarger B (2011). Manipulation of oxygenation and flow-induced shear stress can increase the in vitro yield of platelets from cord blood. Tissue Eng. Part C Methods.

[38] Schlinker AC, Radwanski K, Wegener C, Min K, Miller WM (2015). Separation of in-vitro-derived megakaryocytes and platelets using spinning-membrane filtration. Biotechnol. Bioeng..

[39] Avanzi MP (2016). A novel bioreactor and culture method drives high yields of platelets from stem cells. Transfusion.

[40] Yu Q (2015). DNA-damage-induced type I interferon promotes senescence and inhibits stem cell function. Cell Rep..

[41] Davizon-Castillo P (2019). TNF-alpha-driven inflammation and mitochondrial dysfunction define the platelet hyperreactivity of aging. Blood.

[42] Sun S (2021). Single-cell analysis of ploidy and the transcriptome reveals functional and spatial divergency in murine megakaryopoiesis. Blood.

[43] Shih T-H, Liou WW, Shabbir A, Yang Z, Zhu J (1995). A new k-ϵ eddy viscosity model for high reynolds number turbulent flows. Comput. Fluids.

[44] Oikawa S, Sasaki D, Kikuchi M, Sawamura Y, Itoh T (2013). Comparative in vitro evaluation of apheresis platelets stored with 100% plasma versus bicarbonated Ringer’s solution with less than 5% plasma. Transfusion.

