# Supplementary material for: Defective flow space limits the scaling up of turbulence bioreactors for platelet generation
Source: Commun Eng. 2024 Jun 17;3:77. doi: 10.1038/s44172-024-00219-y (PMC11183101; doi:10.1038/s44172-024-00219-y)
Supplement: Supplementary file 1 — Supplementary Information [file 44172_2024_219_MOESM1_ESM.docx]

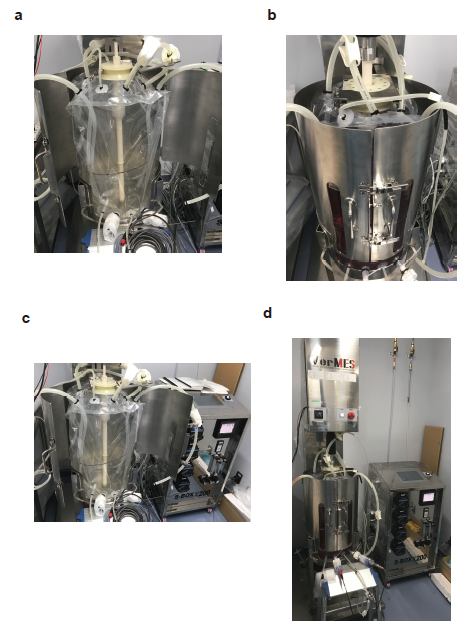


**Supplementary Figure 1:** **Pictures of GMP-grade VerMES50 (two-impeller model).**

Pictures of VerMES 50 with zoom on the single-use polyethylene material tank (a, b) or the wider view (c, d), and with the hatch open (a, c) or closed (b, d).

**
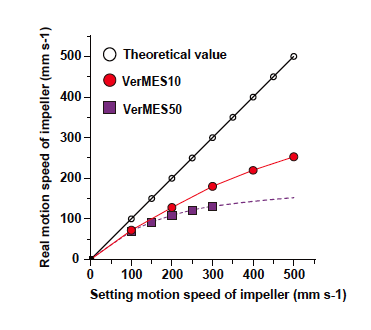
**

**Supplementary Figure 2: Effects of impeller speed on dissociation with the theoretical value in VerMES (two-impeller model).**

Relationship between the setting motion speed and real motion speed of the impeller in VerMES10 and VerMES50. Each VerMES had a different controller system.


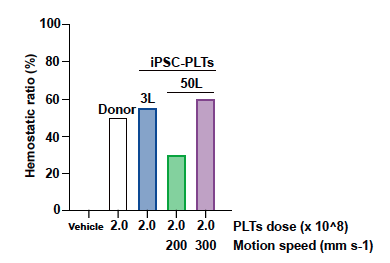


**Supplementary Figure 3: Hemostatic ratio after platelet transfusion in a thrombocytopenia mouse model.**

The hemostatic ratio was evaluated by the number of hemostatic mice per total mice (%). Data includes donor platelets (Donor), iPSC-PLTs produced by VerMES3 (3L), and VerMES50 (50L) (motion speed: 200 and 300 mm s-1). PLTs; platelets, iPSC-PLTs; induced-pluripotent-stem-cell-derived platelets.


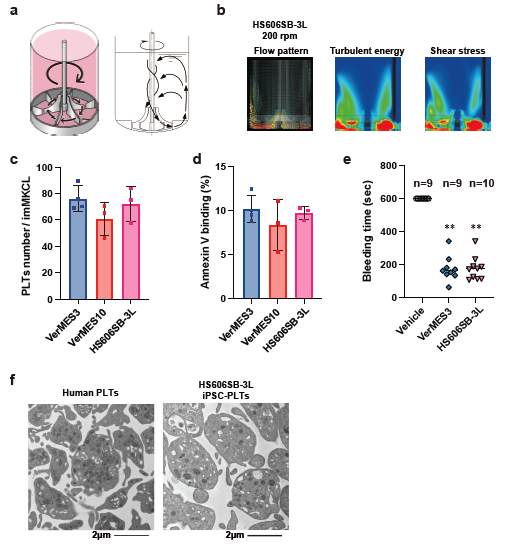


**Supplementary Figure 4: Design and feasibility of new rotation-type reactor, HS606SB**

**a.** Illustration of the HS606SB impeller and wall structure. **b.** A CFD analysis visualizing the flow pattern, turbulent energy, and shear stress of HS606SB-3L. **c.** The production of CD41^+^CD42b^+^ iPSC-PLTs from imMKCLs in VerMES3: Blue bar (N=4), and VerMES10 Red bar (N=3), and HS606SB-3L Pink bar (N=3). Values were taken at Dox-OFF day 6 (mean ± SD). **d.** Annexin V binding quantification by flow cytometry. **e.** Bleeding times after transfusion in a thrombocytopenia mouse model. *：P<0.05, **：P<0.01, Mann-Whitney test vs. vehicle. **f.** TEM images of human PLTs and iPSC-PLTs. iPSC-PLTs were taken at Dox-OFF day 6 from HS606SB-3L. Scale bars: 2 μm. PLTs; platelets, imMKCL; immortalized megakaryocyte cell line, iPSC-PLTs; induced-pluripotent-stem-cell-derived platelets.


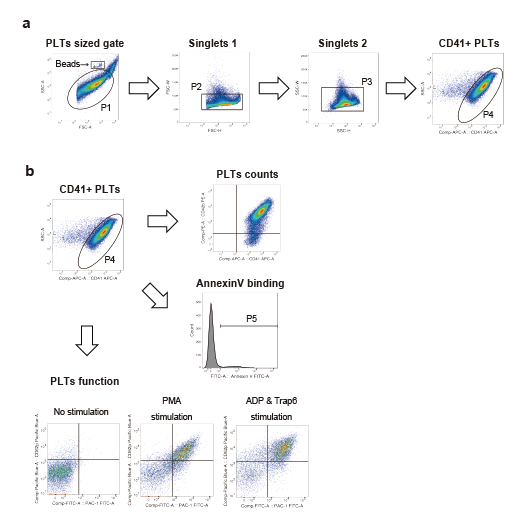


**Supplementary Figure 5: Flow cytometry gating strategies for iPSC-PLTs.**

iPSC-PLTs counted with the aid of absolute counting beads. **b.** Gated CD41+PLTs were used to measure PLTs number counts, AnnexinV binding assay, and PLTs functionality assay. PLTs; platelets, iPSC-PLTs; induced-pluripotent-stem-cell-derived platelets.

| Scale | Speed | Turbulent energy | Shear stress | Kolmogorov scale | Shear rate | Vorticity | Dissipation of energy |
| --- | --- | --- | --- | --- | --- | --- | --- |
| (L) | (rpm) | (m2 s-2) | (Pa) | (μm） | (s-1) | (s-1) | (m2 s-3) |
| 3 | 200 | 0.0105 | 3.52 | 184 | 32.4 | 29.8 | 0.1602 |

**Supplementary Table 1: The CFD simulation of HS606SB-3L model.**

The CFD analysis results show the optimal values of motion speed, turbulent energy, shear stress, Kolmogorov scale (vortex size), shear rate, vorticity, and dissipation of energy for HS606SB.

| Model | Mesh Type | Maximum Mesh Size  [mm] | Number of Meshes  [cells] |
| --- | --- | --- | --- |
| HS606SB-3L | Polyhedral | 4.0 | About 470,000 |
| HS606SB-10L | Polyhedral | 4.0 | About 950,000 |
| VerMES3 | Hexahedral | 1.8 | About 1,100,000 |
| VerMES10 | Hexahedral | 1.8 | About 3,500,000 |
| VerMES50 | Hexahedral &  Polyhedral | 3.6 & 10 | About 660,000 |

**Supplementary Table 2: Mesh parameters of physical flow simulations in bioreactors**

Mesh parameters used in the CFD analysis of each bioreactor in this study are shown.
