# Supplementary material for: Defective flow space limits the scaling up of turbulence bioreactors for platelet generation
Source: Commun Eng. 2024 Jun 17;3:77. doi: 10.1038/s44172-024-00219-y (PMC11183101; doi:10.1038/s44172-024-00219-y)
Supplement: Supplementary file 2 — Description of Additional Supplementary Files [file 44172_2024_219_MOESM2_ESM.pdf]

Description of Additional Supplementary Files

File name: Supplementary Movie 1a, 1b, 1c

Description: The CFD simulation of VerMES Two-impeller model. CFD analysis results showing the optimal values of motion speed, turbulent energy, shear stress, Kolmogorov scale (vortex size), shear rate, vorticity, and dissipation of energy for VerMES3, VerMES10, and VerMES50.

File name: Supplementary Movie 2

Description: The CFD simulation of VerMES50 Three-impeller model. The CFD analysis indicated that three impellers give optimal motion speed, turbulent energy, shear stress, Kolmogorov scale, shear rate, vorticity, and dissipation of energy.
