# Supplementary material for: Defective flow space limits the scaling up of turbulence bioreactors for platelet generation
Source: Commun Eng. 2024 Jun 17;3:77. doi: 10.1038/s44172-024-00219-y (PMC11183101; doi:10.1038/s44172-024-00219-y)
Supplement: Supplementary file 7 — Reporting Summary [file 44172_2024_219_MOESM7_ESM.pdf]

nature portfolio

Corresponding author(s): *Double-blind peer review submissions: write DBPR and your manuscript number here instead of author names.*

Last updated by author(s): *YYYY-MM-DD*

Reporting Summary

Nature Portfolio wishes to improve the reproducibility of the work that we publish. This form provides structure for consistency and transparency in reporting. For further information on Nature Portfolio policies, see our Editorial Policies and the Editorial Policy Checklist.

Statistics

For all statistical analyses, confirm that the following items are present in the figure legend, table legend, main text, or Methods section.

n/a	Confirmed	
☐	☒	The exact sample size (*n*) for each experimental group/condition, given as a discrete number and unit of measurement
☐	☒	A statement on whether measurements were taken from distinct samples or whether the same sample was measured repeatedly
☐	☒	The statistical test(s) used AND whether they are one- or two-sided<br>*Only common tests should be described solely by name; describe more complex techniques in the Methods section.*
☒	☐	A description of all covariates tested
☐	☒	A description of any assumptions or corrections, such as tests of normality and adjustment for multiple comparisons
☐	☒	A full description of the statistical parameters including central tendency (e.g. means) or other basic estimates (e.g. regression coefficient) AND variation (e.g. standard deviation) or associated estimates of uncertainty (e.g. confidence intervals)
☐	☒	For null hypothesis testing, the test statistic (e.g. *F*, *t*, *r*) with confidence intervals, effect sizes, degrees of freedom and *P* value noted<br>*Give P values as exact values whenever suitable.*
☒	☐	For Bayesian analysis, information on the choice of priors and Markov chain Monte Carlo settings
☒	☐	For hierarchical and complex designs, identification of the appropriate level for tests and full reporting of outcomes
☒	☐	Estimates of effect sizes (e.g. Cohen's *d*, Pearson's *r*), indicating how they were calculated

Our web collection on statistics for biologists contains articles on many of the points above.

Software and code

Policy information about availability of computer code

Data collection	Detailed information were provided in the materials and method section. Briefly, BD FACSuite Software was used for collecting FACS Data. Bulk RNA-sequencing was performed using a HiSeq2500 (Illumina) platform.
Data analysis	For Statistical analysis, GraphPad Prism 10.0.2 was used. For FACS analysis, FlowJo (v10) was used. For bulk RNA-seq, TopHat (version 2.1.1) was used to map to the reference genome (UCSC/hg19) with annotation data from iGenomes (Illumina). Gene expression levels were quantified using Cuffdiff (Cufflinks version 2.2.1). Other tools used: FastQC v0.11.8, SAMtools v1.9, BEDtools v2.27.1. For functional profiling: g:Profiler web server; For Gene Set Enrichment Analysis (GSEA): GSEA 4.3.2 software.

For manuscripts utilizing custom algorithms or software that are central to the research but not yet described in published literature, software must be made available to editors and reviewers. We strongly encourage code deposition in a community repository (e.g. GitHub). See the Nature Portfolio guidelines for submitting code & software for further information.

Data

Policy information about availability of data

All manuscripts must include a data availability statement. This statement should provide the following information, where applicable:

- Accession codes, unique identifiers, or web links for publicly available datasets
- A description of any restrictions on data availability
- For clinical datasets or third party data, please ensure that the statement adheres to our policy

The raw sequence data have been deposited in the XXXXXXXXX database under the association number XXXXXXXXX [https://XXXXXXXXX]. The project is also available at the NCBI BioProject [http://www.ncbi.nlm.nih.gov/bioproject/] under the same association number. The processed data sets were deposited with the association codes XXXXXXXX [https://XXXXXXXXXXX]. The other data supporting the findings of this study are available from the corresponding author upon reasonable request.

Human research participants

Policy information about studies involving human research participants and Sex and Gender in Research.

Reporting on sex and gender	n/a
Population characteristics	n/a
Recruitment	n/a
Ethics oversight	n/a

Note that full information on the approval of the study protocol must also be provided in the manuscript.

Field-specific reporting

Please select the one below that is the best fit for your research. If you are not sure, read the appropriate sections before making your selection.

☒ Life sciences ☐ Behavioural & social sciences ☐ Ecological, evolutionary & environmental sciences

For a reference copy of the document with all sections, see nature.com/documents/nr-reporting-summary-flat.pdf

Life sciences study design

All studies must disclose on these points even when the disclosure is negative.

Sample size	No statistical methods were used to predetermine sample size for experiments. The sample sizes were based on those generally employed in the field. All the data include at least three biological replicates
Data exclusions	All the data were included in the analysis.
Replication	All experiments, with the exception of in vivo circulation study, were conducted in a minimum of three biological replicates. All findings were successfully replicated or reproduced at least three times in independent experiments. Although the circulation study was performed once in terms of animal welfare, number of animals in each group was set at least five mice.
Randomization	All the samples were randomly allocated into experimental groups. There is no bias when performing each experiment and collecting data.
Blinding	For in vitro study, the investigators were not blinded since analyses relied on unbiased measurements of quantitative parameters. However, standardized procedures for data collection and analysis were used to prevent bias. For in vivo hemostasis study, administration of the test article and puncture to the tail artery/observation of bleeding time were performed completed separately to avoid any bias.

Reporting for specific materials, systems and methods

We require information from authors about some types of materials, experimental systems and methods used in many studies. Here, indicate whether each material, system or method listed is relevant to your study. If you are not sure if a list item applies to your research, read the appropriate section before selecting a response.

Materials & experimental systems

n/a	Involved in the study
☐	☒ Antibodies
☐	☒ Eukaryotic cell lines
☒	☐ Palaeontology and archaeology
☐	☒ Animals and other organisms
☒	☐ Clinical data
☒	☐ Dual use research of concern

Methods

n/a	Involved in the study
☒	☐ ChIP-seq
☐	☒ Flow cytometry
☒	☐ MRI-based neuroimaging

Antibodies

Antibodies used: For flow cytometric analysis; anti-hCD41-APC (#303710, BioLegend Inc), anti-hCD42b-PE (#303906, BioLegend Inc), anti-hCD62P-Brilliant Violet 421 (#304910, BioLegend Inc), FITC-PAC-1 (#340507, BD Biosciences), FITC Annexin V (#556419, BD Biosciences). For establishment of thrombocytopenia model mice; anti-mouse CD42b antibody (emfret Analytics#R300)

Validation: Antibodies were validated by manufacturers or validated in previous studies. Statements on antibody validation are present on the manufacturer's websites along with relevant references. For flow cytometric analysis, additional validation was done by the use of negative control (control IgG) for FACS. For establishment of thrombocytopenia model mice, depletion of mouse platelet post administration of the antibody was confirmed.

Eukaryotic cell lines

Policy information about cell lines and Sex and Gender in Research

Cell line source(s): imMKCL clone 7

Authentication: https://doi:10.1016/j.cell.2018.06.011

Mycoplasma contamination: Mycoplasma is regularly tested and the cell lines used in this study were negative for mycoplasma contamination

Commonly misidentified lines (See ICLAC register): The cell line used in this study is not present in the registry of commonly misidentified lines

Animals and other research organisms

Policy information about studies involving animals; ARRIVE guidelines recommended for reporting animal research, and Sex and Gender in Research

Laboratory animals: 8-9-week-old male NOG mice (Central Institute for Experimental Animals, Kawasaki, Kanagawa, Japan) or NSG-SGM3 mice (Charles River Laboratories Japan, Yokohama, Japan) were used.

Wild animals: n/a

Reporting on sex: n/a

Field-collected samples: n/a

Ethics oversight: The ethics committees of Kyoto University (Kyoto, Japan)

Note that full information on the approval of the study protocol must also be provided in the manuscript.

Flow Cytometry

Plots

Confirm that:

☒ The axis labels state the marker and fluorochrome used (e.g. CD4-FITC).

☒ The axis scales are clearly visible. Include numbers along axes only for bottom left plot of group (a 'group' is an analysis of identical markers).

☒ All plots are contour plots with outliers or pseudocolor plots.

☐ A numerical value for number of cells or percentage (with statistics) is provided.

Methodology

Sample preparation: For flow cytometry analysis, the iPSC derived platelets were incubated for 30 min with appropriate antibodies at room

Sample preparation	temperature before flow cytometry analysis. Blood samples were diluted with PBS following fixed with ThromboFix (BECKMAN COULTER/#6607130). Then, the samples were treated as mentioned above.
Instrument	BD FACS Lyric
Software	BD FACSuite application were used for collecting FACS Data. Data was then analyzed using FlowJo software v10
Cell population abundance	Cell sorting was not performed in this study.
Gating strategy	The platelets were first gated based on FSC-A/SSC-A. Then, the plateles were gated based on FSC-A/FSC-H and SSC-A/SSC-H to exclude the doublets. The populations in the singlet gate were used for downstream analysis.

☒ Tick this box to confirm that a figure exemplifying the gating strategy is provided in the Supplementary Information.
